# Evaluation of Gastric Conduit Perfusion Using Indocyanine Green Fluorescence During Radical Esophagectomy and Its Correlation With Anastomotic Leak: A Single-Center, Prospective Study

**DOI:** 10.7759/cureus.79989

**Published:** 2025-03-03

**Authors:** Lijin Selvens, Suraj Surendran, Vijay P Abraham, Negine Paul, Samuel Paul Dhinakar Arelly, Yacob Myla, Inian Samarasam

**Affiliations:** 1 Department of General and Upper Gastrointestinal Surgery, Christian Medical College, Vellore, Vellore, IND; 2 Department of Surgery, The Queen Elizabeth Hospital, Woodville South, AUS

**Keywords:** anastomotic leak, esophageal carcinoma, esophagectomy, gastric conduit perfusion, indocyanine green

## Abstract

Background

Anastomotic leak (AL) is a leading cause of morbidity after esophagectomy, with gastric conduit perfusion being a key predictor. The perfusion of the gastric conduit is often analyzed subjectively based on visual inspection, such as the presence of bright bleeding from the resection margin, pulsation of supplying arcade vessels, and tissue color. Indocyanine Green (ICG) fluorescence is a new tool that helps objectively assess the gastric conduit’s perfusion.

Aim

To evaluate the role of ICG fluorescence in assessing gastric conduit perfusion and its correlation with AL.

Methods

This single-center prospective study from a tertiary hospital in South Asia included patients with esophageal cancer undergoing esophagectomy from December 2019 to December 2021. Gastric conduit perfusion was assessed using real-time ICG imaging with a near-infrared ICG camera (KARL STORZ® SE & Co. KG, Tuttlingen, Germany). It was done before and after trans-mediastinal pull-up and compared with visual perfusion assessment. AL was monitored for two weeks postoperatively.

Results

Sixteen patients (Male, 50%; mean age 53.7 years; squamous carcinoma, 81.2%; stage III-IVA, 50%; neoadjuvant treatment, 100%) undergoing minimally invasive esophagectomy (14 McKeown’s, 2 Ivor Lewis) were included. Before and after the trans-mediastinal pull-up, visual assessment revealed "good" perfusion in 15 and 14 patients, respectively. However, according to the ICG-based evaluation, "good" perfusion was seen in just eight and two patients, respectively. Several conduits showing "good" visual perfusion exhibited "sluggish" perfusion on ICG fluorescence. One patient required conduit tip resection due to "poor" ICG perfusion despite a "good" visual assessment. Two patients (13.3%) developed AL, both of whom belonged to a group of five patients with a change in the ICG perfusion pattern of the gastric conduit from "good" to "sluggish" after its trans-mediastinal pull-up. A significant correlation (asymptotic sig. (2-tailed) P=0.022) was observed between real-time changes in the conduit’s ICG perfusion speed between its abdominal and cervical positions and AL occurrence.

Conclusion

ICG fluorescence is a valuable tool for assessing gastric conduit perfusion during esophagectomy, identifying under-perfused conduits more accurately than visual evaluation. The time difference in perfusion speed before and after trans-mediastinal pull-up is critical, and we believe that assessing perfusion at both stages of the operation is crucial to identifying and addressing perfusion-related issues.

## Introduction

Esophagectomy is the principal component of multimodal resectable esophageal cancer (EC) treatment [1]. Anastomotic leak (AL) remains a dreaded complication of esophagectomy, with a reported incidence of up to 35% [2,3]. Reducing the incidence of AL is crucial and remains one of the most challenging issues in surgical practice [4]. The stomach is the most common conduit used following esophagectomy, and the gastric conduit’s perfusion is an essential predictor of anastomotic integrity. The perfusion of the gastric conduit is often analyzed subjectively based on visual inspection, such as bright bleeding from the resection margin, pulsation of supplying arcade vessels, and tissue color. However, a more objective method of assessing gastric conduit perfusion, which can supplement the surgeon’s visual assessment, is needed. Several methods have been tried for intraoperative objective evaluation of gastric conduit perfusion, such as gastric tonometry, laser speckle contrast imaging, spectroscopy, Doppler flowmetry, and angiography [5]. However, these methods were cumbersome, required high-end equipment, were time-consuming, lacked reasonable objectivity, and were not reproducible [6].

Indocyanine green (ICG) fluorescence angiography enables real-time assessment of vascularization and tissue perfusion. This technique has been used in colorectal surgery to assess the perfusion of the colon and in vascular surgery for the intraoperative evaluation of vascular graft patency and diagnostics in peripheral arterial occlusive disease [7]. In esophageal surgery, ICG fluorescence imaging was first introduced to visualize the arterial network around the anastomosis by Shimada et al. [8]. Recent studies have explored the utility of ICG fluorescence in esophageal surgery and indicate that this technology has the potential to predict ALs [6,9]. Its low toxicity, rapid biliary excretion, and excellent safety record have enabled its use in the real-time assessment of vascular perfusion [10]. ICG fluorescence imaging can simultaneously assess the gastric conduit’s macrocirculation and microcirculation, thereby avoiding the ischemic segment of the gastric conduit if present and guiding the placement of the anastomosis. Additionally, it helps in the intraoperative identification of the gastroepiploic vascular arcade [11].

ICG imaging has been used in multiple ways to measure the perfusion of the gastric conduit, both quantitatively and qualitatively. However, only a few studies have assessed the ICG fluorescence signal by measuring the speed of fluorescent coloring after ICG dye administration. This study prospectively assessed the feasibility and safety of ICG fluorescence imaging during esophagectomy. The gastric conduit’s perfusion pattern is assessed by ICG fluorescence using a simple, consistent, reproducible, and easy-to-use method. The relationship between the perfusion patterns and AL is also assessed.

## Materials and methods

This prospective observational study was conducted in the esophagogastric surgery unit of our tertiary care center after obtaining the necessary institutional review board approval (IRB Min. No. 12359/2019). All patients with newly diagnosed resectable EC planned for McKeown’s/Ivor Lewis esophagectomy were recruited between December 2019 and December 2021 after obtaining the necessary written informed consent. The demographic, clinicopathological, biochemical, and neoadjuvant treatment data were collected prospectively from the hospital’s electronic medical records.

All patients received minimally invasive McKeown’s or Ivor-Lewis esophagectomy along with a two-field lymphadenectomy based on their clinical indications. A thoracic epidural catheter was placed in all patients. After creating the gastric conduit but prior to it being pulled up into the neck (McKeown’s) or thorax (Ivor Lewis), the surgeon visually assessed (visual1) its perfusion (i.e., the pulsations, color, and bleeding from cut edges). This was recorded as “good” (bright red bleeding, presence of pulsations, and pink in color), “doubtful” (poor bleeding pattern and/or weak pulsations with or without a change in appearance), or “bad” (no bleeding/altered dark-colored bleeding, absence of pulsations, and dusky appearance).

**Figure 1:**
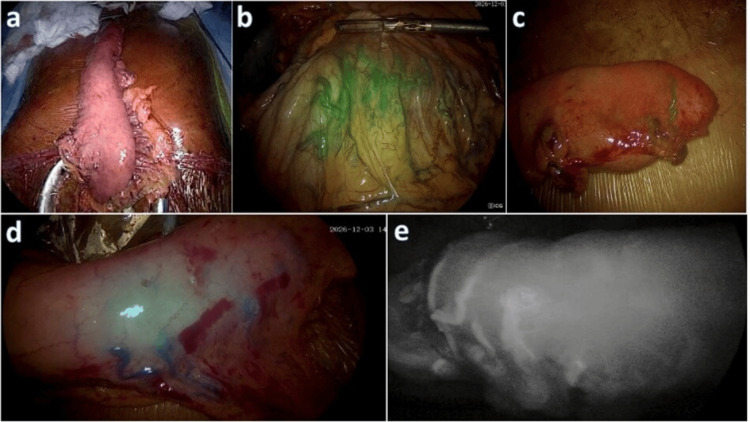
a. Gastric conduit under white light; b. ICG imaging shows fluorescence of the right gastroepiploic artery, demonstrating the conduit’s macrocirculation and microcirculation; c. Overlay mode of ICG imaging showing the perfusion at the tip of the gastric conduit; d. intensity mapping mode; e. monochromatic mode. ICG: Indocyanine Green.

Twenty-five mg of lyophilized sterile powder of ICG (Aurogreen™, Aurolab, Madurai, India, 25mg) was diluted in 10 mL of sterile water for injection and thoroughly shaken for about 3 minutes to allow complete dye dissolution, yielding a 2.5 mg/mL concentration of ICG. Following this, 2 mL (5 mg) of diluted ICG was administered via peripheral venous access and immediately flushed with 10 mL of saline. Simultaneously, real-time imaging of the conduit was done with a near-infrared ICG camera (KARL STORZ® SE & Co. KG, Tuttlingen, Germany), and the images and perfusion pattern findings (ICG1) were recorded. Various ICG imaging modes were utilized to improve the perfusion assessment, as detailed in Figure 1. The ICG readings were always assessed by two surgeons, the primary operating surgeon and a second surgeon on the floor, to prevent bias.

**Figure 2:**
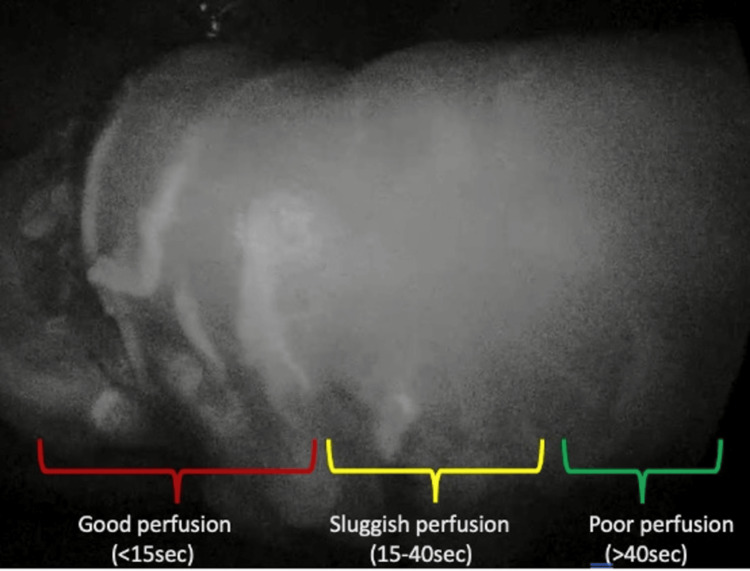
ICG imaging in one of our patients demonstrates the different grades of perfusion areas within the gastric conduit. ICG: Indocyanine Green.

The time for ICG to reach the tip of the gastric conduit after its initial IV injection was measured as an objective measure of perfusion. This time interval was utilized to categorize the perfusion pattern of the gastric conduit at its tip, as detailed below. The areas showing the presence of ICG within 15 seconds and 15-40 seconds from the time of administration were identified as the “good” and “sluggish” perfusion areas, respectively. Those areas showing the presence of ICG at ≥40 seconds were identified as the “poor” perfusion areas (Figure 2), as suggested by Ohio M et al. [12].

The gastric conduit was pulled up to the neck/thorax for anastomosis. Prior to constructing the anastomosis, a second dose of IV ICG was administered, and the conduit perfusion was reassessed using the same method, both visually (Visual2) and using ICG fluorescence (ICG2). Any change in the perfusion pattern of the gastric conduit following its trans-mediastinal pull-up was noted. The half-life of ICG is 3-5 minutes, and its hepatic elimination is completed within 15-20 minutes. Hence, the second dose of ICG was administered about 15 minutes after the first. If the tip of the conduit was found to be ischemic in the neck, confirmed by concordant visual and fluorescence imaging results, the conduit was revised to construct a well-perfused anastomosis. If there was a discrepancy between visual and fluorescence imaging regarding the perfusion of the conduit in the neck, a second surgeon’s opinion was sought, and the decision to revise the conduit was taken based on consensus.

Continuous blood pressure monitoring was performed through an invasive arterial catheter, and the core temperature was monitored constantly to ensure adequate blood pressure and avoid perioperative hypothermia. Active and passive warming techniques were employed throughout the surgery. Patients’ blood pressure and temperature were documented during the perfusion assessments. Additionally, the use of inotropes, duration of surgery, estimated blood loss, and need for blood transfusion were also recorded. Postoperatively, a cervical esophagogastric AL was diagnosed by the presence of erythema, induration, or crepitus of the neck wound with associated salivary discharge and/or signs of mediastinitis. Further imaging was performed to confirm the diagnosis of AL and/or to identify its sequelae. Intra-thoracic leaks were diagnosed when the patient showed signs of mediastinitis and/or pleural sepsis, which was confirmed by an oral contrast study or a computed tomography scan. Patients with AL were managed according to the existing unit protocol.

This study examined the relationship between AL and various perfusion patterns, as determined using visual and ICG assessments. It also evaluated the relationship between the actual time ICG took to reach the tip of the gastric conduit (before and after its trans-mediastinal pull-up) and the occurrence of AL. The incidence of AL during the study period was calculated and compared with the incidence of AL in our unit during a prior period (2015 to 2019) when ICG-based perfusion assessment was not in practice.

Statistical analysis

Continuous variables were documented using mean with standard deviation, median with range, or interquartile range. Categorical variables were reported using frequencies and percentages, and compared using an independent t-test or Mann-Whitney U test. The Chi-square or Fisher exact test was used to find the association between various perfusion-related variables (as detailed above) and the occurrence of an AL. All tests were two-sided at an α=0.05 level of significance. A p-value of <0.05 was considered statistically significant. All statistical analyses were performed using the SPSS, version 21.0 (IBM Corp., Armonk, NY, USA).

## Results

The baseline demographic, clinicopathological characteristics, treatment-related, and intraoperative details are summarized in Table 1.

**Table 1:** Baseline demographic, clinicopathological, treatment-related, and intraoperative details. Values expressed as n (%) or mean ± SD. BMI: Body Mass Index; CCI: Charlson’s Comorbidity Index; cTNM: Clinical Tumor Node Metastasis staging as per AJCC 8th Edition for esophageal cancer; NACT: Neoadjuvant Chemotherapy; NACRT: Neoadjuvant Chemoradiotherapy; ICG: Indocyanine Green.

Variables	N = 16
Age (years)	53.7 ± 7.4
Sex	
Male	8 (50%)
Female	8 (50%)
Diabetes Mellitus	
Yes	2 (12.5%)
No	14 (87.5%)
Charlson’s Comorbidity Index (CCI)	
≤2	4 (25%)
≥2	12 (75%)
Tobacco Consumption	
Yes	9 (56.2%)
No	7 (43.8%)
BMI (Kg/m²)	23.8 ± 4.5
Preoperative Parameters	
Hemoglobin (g/dL)	11.5 ± 1.7
Albumin (mg/dL)	4.2 ± 0.4
Tumor Characteristics	
Location	
Mid thoracic esophagus	6 (37.5%)
Lower thoracic esophagus	6 (37.5%)
Gastroesophageal junction	4 (25%)
Histological Type	
Squamous carcinoma	13 (81.2%)
Adenocarcinoma	3 (18.8%)
cTNM Staging	
Stage I	1 (6.3%)
Stage II	7 (43.7%)
Stage III	6 (37.5%)
Stage IVA	2 (12.5%)
Neoadjuvant Treatment	
NACT	9 (56.2%)
NACRT	7 (43.8%)
Intraoperative Factors	
Type of Esophagectomy	
McKeown’s	14 (87.5%)
Ivor-Lewis	2 (12.5%)
Interoperative Hypothermia	
Yes	0
No	16 (100%)
Intraoperative Inotrope Use	
Yes	10 (62.5%)
No	6 (37.5%)
Intraoperative Transfusions	
Yes	0
No	16 (100%)
Duration of Surgery (min)	501.6 ± 74.7
Estimated Blood Loss (mL)	291.8 ± 174.9
Anastomotic Site Tension	
Yes	0
No	16 (100%)

Patient demographics

Sixteen patients were recruited for the study, comprising eight males and eight females, with an average age of 53.7±7.4 years. The mean body mass index was 23.8±4.5 kg/m². Ten (62.5%) patients had at least one medical comorbidity, and an equal proportion of patients had a history of substance abuse. The mean preoperative hemoglobin, albumin, and absolute neutrophil count were 11.6 gm/dL, 4.08 mg/dL, and 4032 cells/cu.mm, respectively.

Tumor characteristics and details of neoadjuvant treatment

Most of the tumors were squamous cell in origin (81.3%) and were commonly located in the mid or lower thoracic esophagus (37.5% each). Nearly 81% of the patients had ≥cT3 tumors, and about 63% of patients had cN-positive disease. Seven (43.6%) patients received neoadjuvant chemoradiotherapy, and nine (56.3%) received neoadjuvant chemotherapy.

Intra-operative findings

All patients underwent thoraco-laparoscopic esophagectomy. Normothermia was maintained during the perfusion assessments. Ten (62.5%) patients were on single inotropic support (Noradrenaline) during the perfusion assessments and the construction of the anastomosis. All gastric conduits were constructed with adequate intra-abdominal mobilization and without tension at the esophagogastric anastomosis. The hand-sewn anastomotic technique was utilized in all patients. The mean duration of surgery was 501.5±74.7 minutes, and the mean blood loss was 291.8±174.9 mL, with none of the patients requiring any intraoperative blood transfusion.

Assessment of perfusion of the gastric conduit and its association with AL

Among the 16 patients, one patient had an ischemic gastric conduit due to inadvertent injury to the right gastroepiploic artery during the mobilization of the stomach. No pulsations or bright bleeding were noted, and ICG fluorescence confirmed no conduit perfusion. Hence, the gastric conduit was discarded, and the left colon was used as a conduit. This patient was excluded from further analyses of the predictors of an AL. Table 2 details the gastric conduits' perfusion assessments using visual and ICG fluorescence techniques before (Visual1 and ICG1) and after (Visual2 and ICG2) its trans-mediastinal pull-up.

**Table 2:** Perfusion assessment of the gastric conduit before and after pulling up into the thorax/neck. *ICG: Indocyanine Green. Visual1 and ICG1, Visual and ICG perfusion patterns before trans-mediastinal pull-up. Visual2 and ICG2, Visual and ICG perfusion patterns after trans-mediastinal pull-up.

Technique of perfusion assessment N = 15	Good perfusion	Doubtful / Sluggish perfusion	Poor perfusion
Visual1	15	0	0
ICG1	8	7	0
Visual2	14	1	0
ICG2	2	12	1

In one patient who underwent McKeown’s esophagectomy, after trans-mediastinal pull-up of the gastric conduit into the neck, the tip showed good perfusion according to the visual assessment; however, the time taken for ICG dye to reach the tip of the conduit exceeded 40 seconds. Therefore, the decision was made to resect a 2 cm length of the tip of the conduit. The anastomosis was performed in the region with good ICG flow. Since the anastomosis was completed at the 'good' perfusion site following the resection of the ischemic tip, this patient was categorized as having 'good' perfusion for the analysis of AL. This patient did not develop AL.

Our study found a stark difference between visual assessment findings and ICG findings, as detailed in Table 2. Before the trans-mediastinal pull-up of the conduit, good blood flow was seen in all patients by visual assessment vs. eight patients by ICG assessment (100% vs. 53.3%). Doubtful blood flow was not seen in any patient by visual assessment; however, it was observed in seven patients by ICG assessment (46.7% vs. 0%). Following the trans-mediastinal pull-up of the conduit, good blood flow was noted in 14 patients by visual assessment vs. three patients by ICG assessment (93.3% vs. 20%), and doubtful blood flow was seen in one patient by visual assessment vs. sluggish blood flow in 12 patients by ICG assessment (6.7% vs. 80%).

AL developed in two (13.3%) patients; both had undergone McKeown’s esophagectomy. Before this study, our unit’s post-McKeown’s esophagectomy AL rate was 12.7%. The leak rate before and during the study showed no significant difference. Tables 3-4 elaborate on the association between post-pull-up visual (Visual2) and fluorescence-based (ICG2) perfusion patterns of the conduit and AL. Among the 14 patients with 'good' perfusion by visual assessment (Visual2), two (14.3%) experienced a leak. However, these patients’ ICG-fluorescence-based perfusion pattern changed from 'good' (ICG1) to 'sluggish' (ICG2). In 12 patients, the fluorescence-based perfusion pattern of the conduit was 'sluggish' after its trans-mediastinal pull-through; however, only two (16.7%) of these patients developed a leak. Of these 12 patients, none underwent resection of the gastric conduit tip (to modify the anastomotic site) or additional intra-abdominal mobilization to gain more conduit length or supercharging. None of the three patients with a 'good' perfusion pattern following the conduit’s pull-up developed AL. This difference in the incidence of AL between the 'sluggish' perfusion and the 'good' perfusion group was significant (16.7% vs. 0.0%).

**Table 3:** Correlation between visual and ICG-based perfusion patterns of the conduit after its trans-mediastinal pull-up and AL. Visual2: Visual perfusion pattern after trans-mediastinal pull-up. ICG2: ICG perfusion pattern after trans-mediastinal pull-up. ICG: Indocyanine Green; AL: Anastomotic leak.

Type of assessment	Perfusion pattern (N=15)	AL (n = 2)	No AL (n = 13)	Unpaired t-test	P-value
Visual2	Doubtful (1)	0	1	t = 0.98	0.43
	Good (14)	2	12		
ICG2	Sluggish (12)	2	10	t = 1.51	0.27
	Good (3)	0	3		

**Table 4:** Grouped comparison of Visual2 and ICG2 findings with anastomotic leak (AL). ICG: Indocyanine Green.

Visual2	ICG2	AL (n=2)	No AL (n=13)
Doubtful	Sluggish	0	1
Good	Sluggish	2	9
Good	Good	0	3

In five patients (33.3%), the conduit’s ICG-based perfusion pattern changed from 'good' to 'sluggish' following trans-mediastinal pull-up. The two patients with AL belonged to this group, which had a change in its ICG-based perfusion pattern after trans-mediastinal pull-up. In contrast, they were considered part of the 'good' perfusion group based on visual assessment (Visual2) alone.

**Figure 3:**
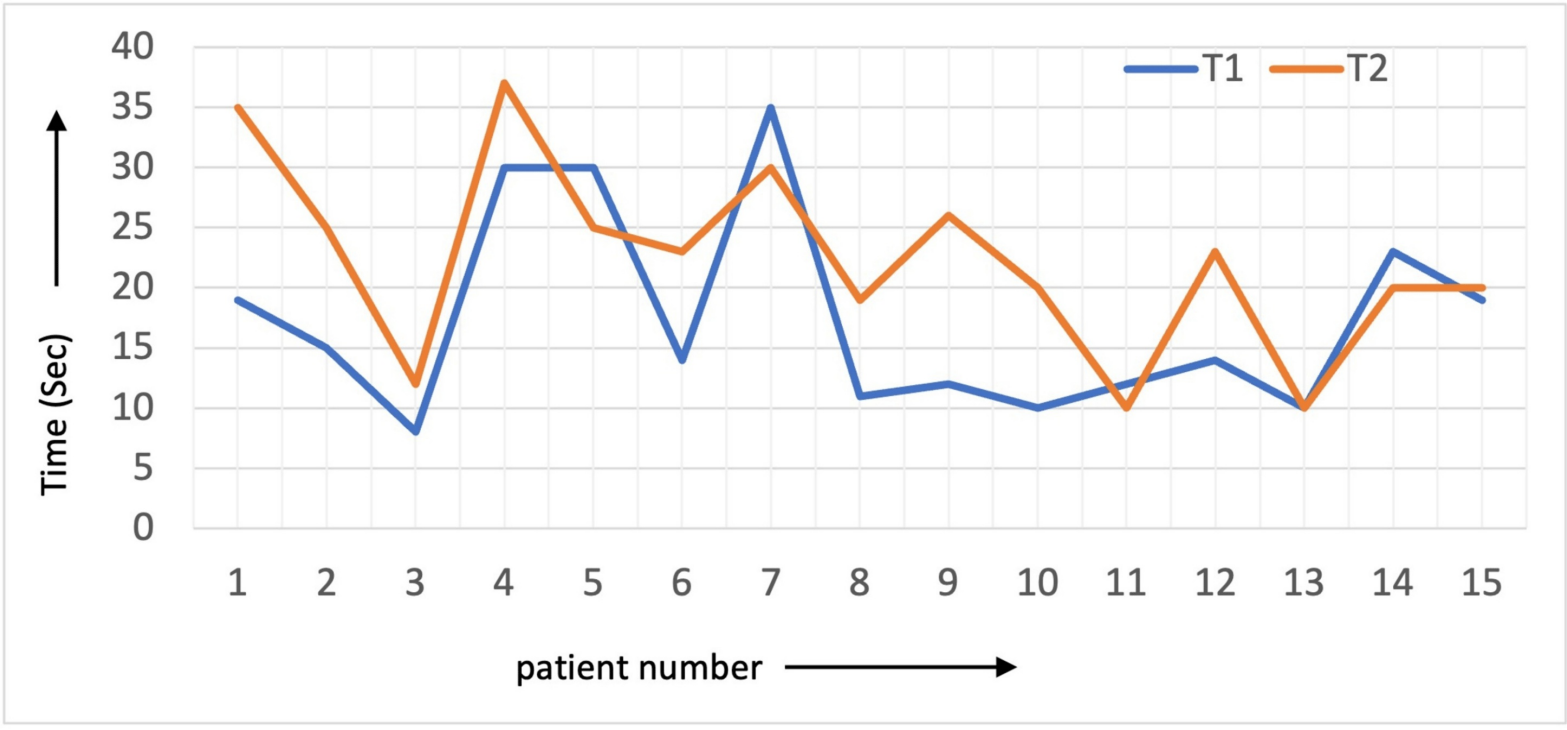
Time taken for ICG to reach the tip of the conduit before (T1) and after (T2) its pull-up. ICG: Indocyanine Green.

The time taken for ICG to reach the tip of the conduit is shown in Figure 3, where T1 was the time taken before, and T2 was the time taken after the trans-mediastinal pull-up. We analyzed the relationship between the time ICG took to reach the conduit tip and the occurrence of AL. In patients with AL, the mean T1 was 12 ± 2 sec, and the mean T2 was 21.5 ± 1.5 sec. In patients who did not have AL, the mean T1 was 18.23 ± 8.8 sec and the mean T2 was 23.3 ± 7.5 sec.

We also calculated the difference in the time the ICG dye takes to reach the conduit tip (i.e., T2-T1) and its relationship with AL. The overall mean change in time for ICG to reach the tip of the conduit was 4.8 ± 6.7 seconds. On further analysis with the NPar test - Wilcoxon signed-rank test, this time interval (T2-T1) significantly correlated with AL; the higher the difference, the greater the likelihood of AL (Table 5). The mean change in time for ICG to reach the conduit tip (T2-T1) was higher in patients with AL than in those without AL (9.5 sec vs. 5.1 sec).

**Table 5:** NPar test between the change in time taken for ICG to reach the tip before and after pulling up into the thorax/neck (T2-T1) and AL. NPar: Non-parametric test; ICG: Indocyanine Green; AL: Anastomotic leak.

Wilcoxon signed-rank test	(T2-T1) seconds
Z score	-2.294
Asymptotic sig. (2-tailed)	0.022

AL and its relationship with other intraoperative and perioperative factors

As detailed in Table 6, the preoperative use of tobacco, levels of hemoglobin and albumin, type of neoadjuvant therapy, intraoperative use of inotropes, and anastomotic technique had no association with the occurrence of a leak.

**Table 6:** Anastomotic leak and its relation with other intraoperative and preoperative factors. Values expressed as n (%) or mean ± SD. CCI: Charlson’s Comorbidity Index; ANC: Absolute Neutrophil Count; SCC: Squamous Cell Carcinoma.

Variables	No AL (n=13)	AL (n=2)	t-statistic (t value)	P-value
Age (years)	53.3 ± 7.2	59.5 ± 6.5	1.41	0.18
Sex				
Male	7 (46.7%)	1 (6.7%)	11	0.06
Female	6 (40.0%)	1 (6.7%)		
Charlson’s Comorbidity Index (CCI)				
≤2	3 (20.0%)	0	2.2	0.27
≥2	10 (66.7%)	2 (13.3%)		
Tobacco Consumption				
Yes	8 (53.3%)	1 (6.7%)	3.67	0.17
No	5 (33.3%)	1 (6.7%)		
Hemoglobin (g/dL)	11.6 ± 1.7	11.4 ± 1.8	1.02	0.32
Albumin (mg/dL)	4.1 ± 0.4	4.05 ± 0.5	1.25	0.24
Absolute Neutrophil Count (ANC) (per mm³)	3991.4 ± 2131.3	4316 ± 956.0	0.36	0.74
Type of Cancer				
Squamous Cell Carcinoma (SCC)	10 (66.7%)	2 (13.3%)	1.45	0.28
Adenocarcinoma	3 (20.0%)	0		
Pre-op CT Staging				
Stage I	1 (6.7%)	0	2.32	0.059
Stage II	5 (33.3%)	2 (13.3%)		
Stage III	5 (33.3%)	0		
Stage IVa	2 (13.3%)	0		
Neoadjuvant Treatment				
Neoadjuvant Chemotherapy (NACT)	8 (53.3%)	1 (6.7%)	3.67	0.17
Neoadjuvant Chemoradiotherapy (NACRT)	5 (33.3%)	1 (6.7%)		
Inotrope Used Intra-op				
Yes	8 (53.3%)	1 (6.7%)	3.67	0.17
No	5 (33.3%)	1 (6.7%)		
Temperature				
Normothermic (36.5-37.5°C)	13 (86.7%)	2 (13.3%)	1	0.5
Hypo/hyperthermic (<36.5/>37.5°C)	0	0		
Type of Esophagectomy				
McKeown’s Esophagectomy	10 (66.7%)	2 (13.3%)	1.67	0.34
Ivor Lewis Esophagectomy	2 (13.3%)	0		
Duration of Surgery (min)	498.5 ± 81.8	502.5 ± 22.5	0.14	0.89

## Discussion

The perfusion of the gastric conduit plays a crucial role in minimizing the risk of AL following esophagectomy. Karampinis I et al. (2017) classified the gastric conduit into two regions: a well-perfused area, called the 'optizone,' and a poorly perfused area. They found that placing the anastomosis in the 'optizone' resulted in a significantly lower rate of AL [13]. Therefore, identifying well-perfused areas of the gastric conduit is essential for constructing the esophagogastric anastomosis in esophagectomies. Traditional methods of conduit perfusion assessment rely on visual cues, such as the color of the conduit. However, efforts have been made to introduce more objectivity into this process [14]. Due to its availability, ICG fluorescence is emerging as a valuable tool for evaluating gastric conduit perfusion. While ICG fluorescence enhances the objectivity of perfusion assessments, qualitative and quantitative fluorescence measurements are still being refined. de Groot EM et al. (2023) measured the time taken for the maximum intensity of ICG in the conduit tip using PINPOINT-assisted technology. They showed that in patients with AL, it took longer for the ICG to reach maximum intensity in the gastric conduit [15]. Nerup N et al. (2020) quantified gastric conduit perfusion using a previously published algorithm, revealing different locations for better placement of anastomosis [16]. However, these methods require specialized, costly equipment and software that are not easily accessible. We devised a simple and reproducible method to evaluate the gastric conduit perfusion with ICG and studied its association with AL. In our study, ICG fluorescence imaging was done in real-time, and none of the patients had any adverse reactions.

Most studies evaluating ICG fluorescence have assessed the perfusion before the trans-mediastinal pull-up of the conduit. Hong ZN et al. (2022) and Kitagawa H et al. (2018) used ICG imaging twice, once before and once after constructing the gastric conduit [17,18]. However, to the best of our knowledge, no studies have analyzed ICG perfusion patterns both before and after the trans-mediastinal pull-up of the gastric conduit. We believe that assessing perfusion at both stages is crucial, as passage through the narrow mediastinal space can further reduce tissue perfusion. Moreover, modifications to the conduit can be made based on the observed perfusion patterns or changes in the speed of ICG fluorescence. This approach would also alert the surgeon to assess the tension, compression, and twist of the conduit.

The perfusion of the conduit was visually assessed as 'good' in 15 patients before it was pulled up through the mediastinum and in all but one patient after the pull-up. However, the ICG-based perfusion pattern of the conduit was rated as 'good' in only eight patients after its preparation in the abdomen and in just two patients after it was pulled trans-mediastinal for anastomosis. The 'sluggish' perfusion zones were better identified with ICG fluorescence imaging than with the surgeon’s visual assessment alone. This highlights the value of ICG fluorescence in detecting perfusion problems at the gastric conduit tip both before and after its pull-up. In clinical practice, when ICG-based perfusion shows a sluggishly perfused conduit in the abdomen, surgeons may address this by resecting the poorly perfused or sluggish tip of the conduit, provided there is sufficient conduit length. Techniques like Kocherisation can help gain an extra 2-3 cm of conduit length. Other methods for lengthening the conduit include creating a pedunculated gastric tube with a duodenal transection that can extend the gastric conduit by an additional 10-15 cm without compromising its macrovascular supply [14]. A change in perfusion pattern from 'good' to 'sluggish' or 'poor' after the conduit is pulled up should raise concerns for potential conduit compression, twisting, or excessive tension. These issues should be thoroughly checked, and corrective measures should be taken before constructing the anastomosis. In one of our patients, the conduit’s visual assessment in the neck was good, but ICG fluorescence showed poor perfusion; hence, the ischemic conduit tip was resected, and anastomosis was performed without further conduit mobilization.

Does the use of ICG fluorescence-based perfusion assessment help reduce the incidence of AL? In our study, AL developed in two patients who underwent McKeown’s esophagectomy, resulting in a leak rate of 13.3%, consistent with the leak rates reported in the literature, ranging from 0% to 35% [2]. A recent meta-analysis comparing the incidence of AL following esophagectomy with and without ICG fluorescence found a significant reduction in leak rates in the ICG group (5.7% vs. 22.9%) [19]. Another study done by Karampinis I et al. also showed low AL rates with the use of ICG as compared to without (8% vs 18%) [13]. Kumagai Y et al. (2018) showed that poor perfusion of the gastric conduit tip is associated with a higher AL rate, attributing this to the lack of collateralization of the right-to-left gastroepiploic artery [20]. Zehetner J et al. (2015) conducted one of the largest studies using ICG to evaluate conduit perfusion and found that patients with anastomoses placed in well-perfused areas proximally had significantly lower AL rates compared to those with anastomoses placed distally (2% vs. 45%; p <0.0001) [21]. In our study, five patients (33.3%) showed a change in their ICG perfusion pattern from 'good' to 'sluggish' following the trans-mediastinal pull-up, and two of these patients developed AL. However, the visual assessment of perfusion in the neck of these two patients (Visual2) was documented as 'good.' We did not resect the sluggishly perfused areas in these patients. No compression or twist of the conduit was found in these patients, and the conduit length was adequate. In hindsight, we might have considered resecting the sluggish tip, mobilizing the conduit further, and anastomosing it in a fluorescence-rich area. In three patients with good visual and ICG-based perfusion, no AL developed. One patient had 'poor' perfusion at the gastric conduit tip after the trans-mediastinal pull-up, as assessed by ICG, and we resected 2 cm of the tip to construct the anastomosis in a well-perfused zone. This patient did not develop AL. A similar outcome was observed by Campbell C et al. (2015), who reported a reduction in AL incidence from 20% to 0% after using ICG-based evaluation to modify the gastric conduit tip [22]. In three patients with both good visual and ICG-based perfusion, no AL developed.

Only a limited number of studies have explored the correlation between the time it takes for ICG to reach the conduit tip and the risk of AL. Koyanagi K et al. (2022) showed that the risk of AL was higher in patients with slower ICG speed. Kumagai Y et al. conducted two studies to evaluate the efficacy of using ICG travel time to assess conduit perfusion. They found that placement of anastomosis in areas with 'good' perfusion (time taken by ICG <60 sec) had a lower AL rate as compared to those with 'poor' perfusion (time taken by ICG >60 sec) [4]. Luo RJ et al. (2021) reported similar findings, describing 'poor' perfusion as ICG reaching the conduit tip in more than 60 seconds [23]. Ohi M et al. (2017) showed that the ICG transit time in most of their study patients was 15-40 seconds [12]. In a recent report by Noma K et al. (2018), the time for ICG to reach the conduit tip was <30 seconds in most patients [24]. While there is no consensus on an acceptable single cut-off value for the time ICG takes to reach the tip of the gastric conduit in literature, it is clear that the longer the time taken, the higher the risk of a leak. We found that in almost all our patients, the time ICG took to reach the conduit tip was <40 seconds, including in the two patients with an AL. However, we also noted that the higher the change in time for ICG to reach the gastric conduit tip between its abdominal and thoracic/cervical positions, the higher the likelihood of AL.

These findings highlight the adjunct role of ICG in assessing gastric conduit perfusion. In our opinion, both the ICG perfusion pattern and the time it takes for ICG to reach the conduit tip, along with the time difference in perfusion speed before and after the pull-up, can aid the surgeon in determining whether conduit modification is needed and ensure placement of anastomosis in a well-vascularized area. If visual perfusion appears satisfactory but ICG reveals persistent sluggish perfusion, with a significant time difference in fluorescence reaching the conduit tip between its abdominal and thoracic/cervical positions, the surgical team should consider resecting the conduit tip and execute additional techniques to ensure a sufficient conduit length.

We also examined the role of various preoperative and intraoperative factors associated with AL. We found no single factor with a significant association, a finding consistent with the observations of Nerup N et al. (2017) [16]. However, Van Daele E et al. (2019) identified preoperative factors such as age and male sex as being associated with AL, while preoperative albumin and BMI were not [25]. Kassis ES et al. (2013) found that diabetes, smoking, and cardiovascular disease can impair conduit microvascularity and are linked to post-esophagectomy leaks. He also noted that patients receiving neoadjuvant radiochemotherapy did not have a higher risk of AL, a finding similar to ours [26]. It is well-established that AL following esophagectomy is multifactorial [25-27]. Therefore, modifying various perioperative and intraoperative risk factors could help further reduce the risk of AL.

The strengths of our study include its prospective design, clear definition of study variables and outcomes, and the consecutive recruitment of patients. All esophagectomies were performed for malignant indications, which helped reduce baseline heterogeneity. Our study had a few limitations. First, it does not determine whether modifying the anastomotic site or reshaping the conduit based on the ICG perfusion pattern can prevent ALs, as there was no control group. Second, the sample size was small since the study was conducted as part of a pilot study. A compromise in the venous drainage of the gastric conduit can result in AL. Although ICG fluorescence is effective in assessing the arterial blood flow of the conduit, it does not evaluate its venous drainage. As a result, the role of venous insufficiency contributing to AL could not be determined in our study, which represents the third limitation of this research. While ICG offers more objectivity than visual assessment, the perfusion evaluation remains subjective and may vary between surgeons. Despite efforts to minimize this subjectivity, inter-observer variations may have occurred, representing another limitation of this study.

## Conclusions

ICG fluorescence imaging is a simple and novel technique for evaluating gastric conduit perfusion during esophagectomy. It can be performed in real-time and is considered safe. This method serves as a valuable adjunct for surgeons to identify under-perfused conduits more accurately than visual evaluation alone. The ICG perfusion pattern, the speed at which ICG reaches the conduit tip, and the time difference in perfusion speed before and after trans-mediastinal pull-up are critical factors in assessing the perfusion of the gastric conduit. We believe that assessing perfusion at both stages of the operation is crucial for identifying and addressing perfusion-related issues.
